# Using autoantibody signatures to define cancer risk in dermatomyositis

**DOI:** 10.1172/JCI156025

**Published:** 2022-01-18

**Authors:** Jessica L. Turnier, J. Michelle Kahlenberg

**Affiliations:** 1 Department of Pediatrics, Division of Rheumatology,; 2 Department of Internal Medicine, Division of Rheumatology,; 3 Department of Dermatology, University of Michigan, Ann Arbor, Michigan, USA.

## Abstract

Dermatomyositis is an idiopathic inflammatory myopathy with a highly heterogeneous disease course. Although there is a known increase in cancer risk surrounding the time of dermatomyositis diagnosis, the mechanisms driving this increased risk are not well understood. Further, there are no current standardized cancer screening guidelines for dermatomyositis patients. In this issue of the *JCI*, Fiorentino, Mecoli, et al. discovered additional autoantibodies in patients with dermatomyositis and anti–TIF1-γ autoantibodies, a known risk factor for malignancy. They observed a decreased cancer risk with an increasing number of autoantibodies. Importantly, these findings indicate that more detailed autoantibody phenotyping at diagnosis might better predict cancer risk and also suggest that diversity and kinetics of the host immune response might influence cancer development.

## Myositis-specific autoantibodies and a relationship to cancer

The discovery of myositis-specific autoantibodies (MSAs) has been an important step toward understanding disease heterogeneity and paving the way for personalized medicine in dermatomyositis (DM). MSAs associate with distinct clinical phenotypes (1), and it is now possible to utilize MSAs to stratify patients with DM for risk of potential disease complications and associated outcomes. Notably, there is an increased risk of cancer with specific MSAs, most commonly anti–transcription intermediary factor 1-γ (TIF1-γ) (2, 3).

The cancer-DM relationship is well recognized, and cancer-associated myositis (CAM) affects up to 30% of DM patients, most often within the first year of diagnosis. Although multiple clinical variables, in addition to MSA status, associate with risk or protection from the development of cancer in DM (4), we do not yet understand the mechanisms linking MSAs or clinical phenotypes to the onset of cancer. Moreover, even within the subgroup of patients with DM considered at highest risk for cancer based on MSA and clinical status, these patients may remain cancer free. Thus, there exists an unmet need to improve cancer risk stratification in DM in order to develop targeted cancer screening guidelines (for which none are currently standardized; ref. 5) and improve patient care.

## Autoantibody signatures in DM associate with cancer risk

In a retrospective, multicenter cohort study, Fiorentino, Mecoli, et al. (6) identified autoantibodies present in patients with anti–TIF1-γ–positive DM that conferred protection against cancer development, with a dose-response relationship between increasing number of autoantibodies and decreasing cancer frequency (6). The authors first used plasma from 36 DM patients either with or without a cancer diagnosis (*n =* 18 per group) to immunoprecipitate proteins from melanoma cell lysates. The investigators discovered an increased number of immunoprecipitated proteins in patients with DM but without cancer, thereby noting an overall greater target autoantigen diversity in the absence of associated cancer. Next, they performed mass spectrometry sequencing on the immunoprecipitates from five of the anti–TIF1-γ–positive patients without cancer and identified 23 candidate autoantigens, subsequently validating 10 of these targets that were unique to DM patients as compared with healthy controls (6).

Fiorentino, Mecoli et al. screened for these 10 candidate autoantibodies in two independent cohorts of subjects with DM from Stanford (*n =* 110) and Johns Hopkins (*n =* 142), identifying at least one autoantibody in 50% of all patients and defining cell division cycle and apoptosis regulator protein 1 (CCAR1) as the most common autoantigen (6). Antibodies recognizing CCAR1 were present in 32% of patients with DM that were positive for anti–TIF1-γ in both cohorts and were found in only 1.5% of patients with scleroderma who possessed anti-POLR3A autoantibodies, suggesting good disease specificity. Patients with the sole presence of anti–TIF1-γ had a two- to four-fold higher incidence of cancer, whereas anti-CCAR1 antibodies were negatively associated with cancer within three years of diagnosis (OR 0.27, *P =* 0.05 [Stanford]; OR 0.13, *P =* 0.008 [Johns Hopkins]). If cancer was ultimately diagnosed in patients with DM and anti-CCAR1 autoantibodies, cancers were of a lower stage and occurred further from DM diagnosis (4.3 vs. 0.87 years). Interestingly, anti-CCAR1 autoantibodies were specific to the anti–TIF1-γ–positive subset, as they were found in only 1 out of 172 patients who had DM but were negative for anti–TIF1-γ. The tight relationship between these autoantigens was further enhanced by data that show that CCAR1 and TIF1-γ associate within the same molecular complex (6).

## Further study and insight into the DM-cancer relationship

Fiorentino, Mecoli, et al. (6) also generate an important discussion related to understanding the body’s natural immune regulation of cancer and specifically the phases of cancer immunoediting (7) in the context of their data. The investigators speculate that greater diversity of the immune response in anti–TIF1-γ–positive patients with multiple autoantibodies may be protective against cancer through increased time spent in the equilibrium phase of immunoediting, allowing a greater likelihood of immune-mediated elimination of precancerous or cancerous cells. This process may reflect a broader mechanism relevant to multiple autoimmune diseases, as scleroderma patients with anti-RPA194 in addition to anti-POLR3A also have decreased cancer risk (8). The individual microenvironment in which immunoediting occurs also likely contributes to outcome, and it is important to consider shifts in this microenvironment in the context of ongoing and changing immunomodulatory therapy. It is known that immune cell type, density, and spatial orientation within tissue at the site of cancer influence survival and response to therapy, in particular immune checkpoint inhibitors (9). In a given individual with DM, the process of immunoediting is also potentially altered by the stage and type of host immune response and consideration of time from DM diagnosis and ongoing treatment effects. Another remaining question is whether some cases of CAM may actually arise during the process of cancer immunoediting.

Given that TIF1-γ is a nuclear antigen, it is possible that pathways in cell death and damage expose this antigen to the immune response (10), perhaps in the context of cancer cell death. Other hypotheses for the presence of anti–TIF1-γ antibodies include increased baseline expression of myositis antigens, including TIF1-γ, in DM muscle, predominantly in regenerating cells (11, 12). The skin could also play a role. Patients with DM who possess anti–TIF1-γ autoantibodies have a more severe skin disease phenotype and, notably, photosensitivity. Interestingly, patients with juvenile DM (JDM) who experienced increased UV radiation exposure in the month prior to disease onset had a higher likelihood of having anti–TIF1-γ autoantibodies (13), suggesting a potential role for skin inflammation in modifying disease phenotype and MSA development. Clustering of systemic lupus erythematosus autoantigens has previously been demonstrated on the surface of apoptotic keratinocytes (14), and a similar mechanism could operate in DM keratinocytes. It has already been shown that Mi-2, another MSA, shows increased expression in keratinocytes for up to 16 hours after UV exposure (15). Given that CCAR1 was discovered through testing antigens isolated from a melanoma cell line, it is intriguing to speculate that skin cancers may be an important contributor to this MSA. The authors acknowledge that they have yet to test other cancer lines for additional antibody specificities (6).

Other functions of TIF1-γ include its role as an E3 ubiquitin-protein ligase and nuclear protein that likely acts as a transcriptional repressor. TIF1-γ has various roles in inflammation as well. In macrophages, it is necessary for *IFNB1* transcription shutdown (16), and it also regulates NLRP3 inflammasome activation (17). In a mouse model of inflammatory bowel disease, TIF1-γ expression in monocytes/macrophages was shown to be essential to resolve inflammation (18). Importantly, TIF1-γ can inhibit tumor growth and metastasis (17). Expression of TIF1-γ varies in different tumor types (17), but the mechanisms by which it functions in malignancy need further study. Whether antibodies against TIF1-γ reflect nonspecific cancer-driven immune activation or whether they have a functional role in modulating the mechanisms by which TIF1-γ is involved in cancer growth regulation remains unknown. Similarly, how anti-CCAR1 antibodies modulate cancer growth needs further investigation. Interestingly, CCAR1 gene expression levels have been demonstrated to associate with cancer survival, although results vary with cancer type; low CCAR1 expression levels correlate with increased survival in liver and renal cancers and the opposite has been shown in ovarian cancers (19). Of note, individuals with anti–TIF1-γ^+^ DM as compared with anti–TIF1-γ^–^ DM more commonly have ovarian cancer as compared with other cancer types (20).

## MSA clinical phenotypes in children and adults

Although TIF1-γ is one of the most frequently encountered MSAs in JDM (21), TIF1-γ is almost never associated with cancer development in children. In the absence of atypical clinical features suggestive of underlying malignancy, pediatric rheumatologists do not routinely screen for cancer in JDM. Children with anti–TIF1-γ autoantibodies demonstrate more severe skin disease as compared with adults (22) and are more likely to have a chronic disease course (23). Anti–TIF1-γ autoantibodies from both children and adults target the same autoantigen in K562 leukemic cells (22), although tissue-specific differences and potential unique protein epitopes are unknown. It is possible that differential skewing of the immune response related to patient age and other factors plays a role in disease phenotype (24), disease course, and potential development of cancer. Further, it is important to understand immune responses that are protective against cancer development and study anti–TIF1-γ–positive JDM patients.

Comparison of disease immunophenotypes in children versus adults could lend insight into protective mechanisms. Screening a pediatric cohort of patients with JDM for anti-CCAR1 and other autoantibodies could serve as a first step to highlight similarities and differences in the age-specific host immune response in myositis. It would be interesting to determine whether anti–TIF1-γ–positive DM patients who do not develop cancer demonstrate an immunophenotype more similar to patients with JDM. It would also be interesting to study whether skewing of the immune response toward the development of calcinosis protects against cancer development. Notably, children more commonly develop calcinosis than adults with DM (25) and most frequently in the presence of antibodies against nuclear matrix protein-2 (NXP2) (26), another MSA that has been associated with cancer in DM (27).

In summary, Fiorentino, Mecoli, et al. (6) find additional biomarkers that may fine-tune our understanding of cancer risk in adult DM patients. Still, more importantly, the study adds insight into the intricate relationship between cancer and autoimmunity. Further investigation of the role of TIF1-γ, CCAR1, and other immune targets in individuals with DM is warranted.

## Version 1 01/18/2022

Electronic publication
